# *geneHummus*: an R package to define gene families and their expression in legumes and beyond

**DOI:** 10.1186/s12864-019-5952-2

**Published:** 2019-07-18

**Authors:** Jose V. Die, Moamen M. Elmassry, Kimberly H. LeBlanc, Olaitan I. Awe, Allissa Dillman, Ben Busby

**Affiliations:** 1 Department of Genetics ETSIAM, University of Córdoba, Córdoba, Spain; 2 National Center for Biotechnology Information, National Library of Medicine, National Institutes of Health, 8600 Rockville Pike, Bethesda, MD 20894 USA; 3 Department of Biological Sciences, Texas Tech University, TX, Lubbock, 79409 USA; 4 National Institute on Drug Abuse, National Institutes of Health, 6001 Executive Blvd, Bethesda, MD 20892 USA; 5 Department of Computer Science, University of Ibadan, Ibadan, Nigeria

**Keywords:** Bioinformatics, Genome annotation, Gene family, Plant breeding, Rstat, Pipeline, Phylogenetic tree, RefSeq, SPARCLE

## Abstract

**Background:**

During the last decade, plant biotechnological laboratories have sparked a monumental revolution with the rapid development of next sequencing technologies at affordable prices. Soon, these sequencing technologies and assembling of whole genomes will extend beyond the plant computational biologists and become commonplace within the plant biology disciplines. The current availability of large-scale genomic resources for non-traditional plant model systems (the so-called ‘orphan crops’) is enabling the construction of high-density integrated physical and genetic linkage maps with potential applications in plant breeding. The newly available fully sequenced plant genomes represent an incredible opportunity for comparative analyses that may reveal new aspects of genome biology and evolution. The analysis of the expansion and evolution of gene families across species is a common approach to infer biological functions. To date, the extent and role of gene families in plants has only been partially addressed and many gene families remain to be investigated. Manual identification of gene families is highly time-consuming and laborious, requiring an iterative process of manual and computational analysis to identify members of a given family, typically combining numerous BLAST searches and manually cleaning data. Due to the increasing abundance of genome sequences and the agronomical interest in plant gene families, the field needs a clear, automated annotation tool.

**Results:**

Here, we present the *geneHummus* package, an R-based pipeline for the identification and characterization of plant gene families. The impact of this pipeline comes from a reduction in hands-on annotation time combined with high specificity and sensitivity in extracting only proteins from the RefSeq database and providing the conserved domain architectures based on SPARCLE. As a case study we focused on the auxin receptor factors gene (ARF) family in *Cicer arietinum* (chickpea) and other legumes.

**Conclusion:**

We anticipate that our pipeline should be suitable for any taxonomic plant family, and likely other gene families, vastly improving the speed and ease of genomic data processing.

**Electronic supplementary material:**

The online version of this article (10.1186/s12864-019-5952-2) contains supplementary material, which is available to authorized users.

## Background

**Fig. 1:**
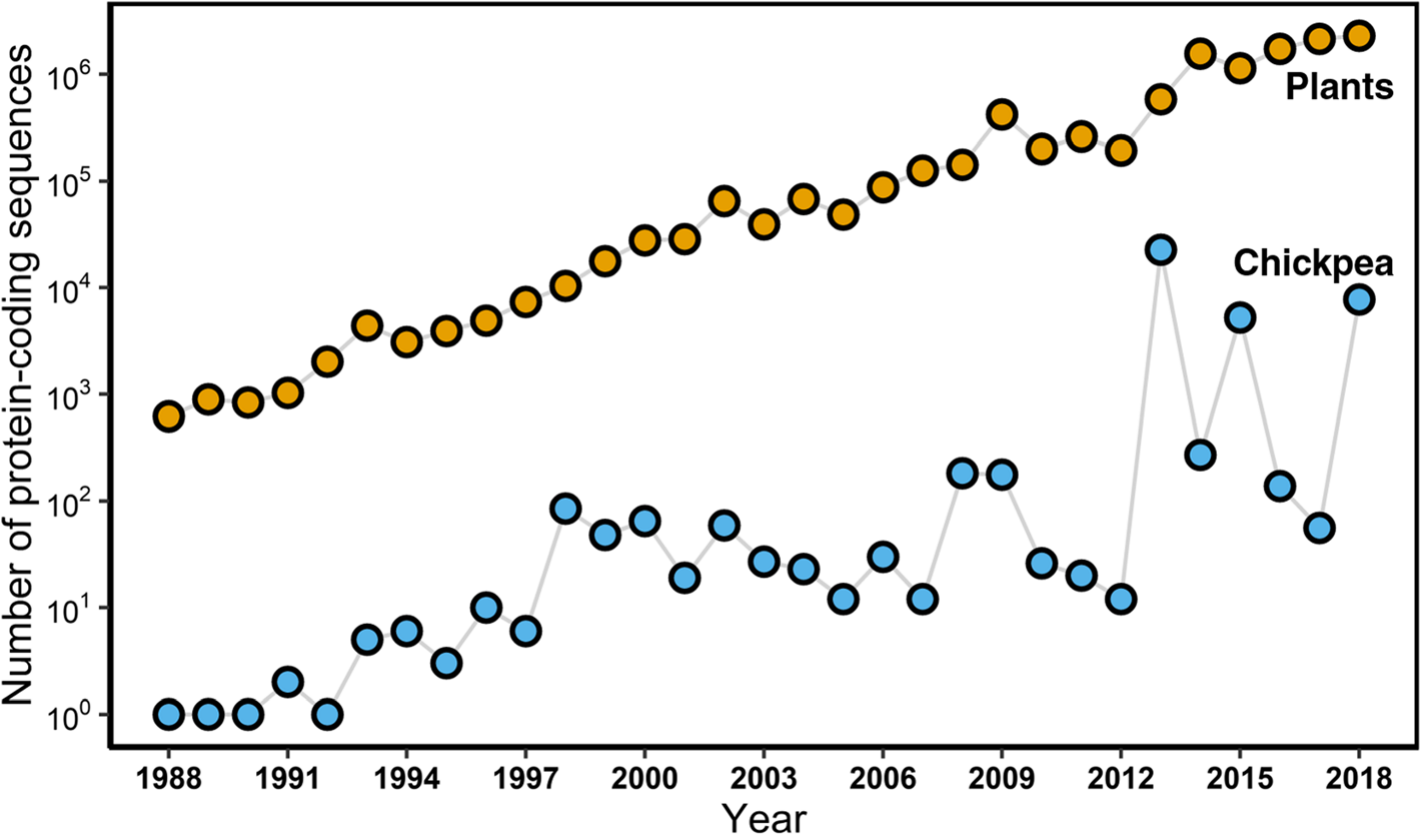
Statistics on the number of protein-coding sequences deposited in NCBI every year**.** Number of plants and chickpea protein-coding sequences deposited in NCBI over the past 3 decades

By using next-generation sequencing (NGS) technology, researchers have massively increased the number of nucleotide sequences deposited in public databases [1]. This had revolutionized numerous fields including the plant sciences (Fig. 1). However, a bottleneck in the field of plant sciences is the annotation of the protein sequences and the characterization of their functions. Identifying the function of important proteins can be used to improve agronomic performance, like altering resistance or tolerance of plants to specific environmental stressors such as drought or heat. One approach to infer the function of an unknown protein is to identify conserved sequences among proteins with known function, which can be useful to the extent that homology can imply conserved biochemical function [2].

The plant hormone auxin (indole-3-acetic acid) is a key regulator of virtually every aspect of plant growth and development [3]. As a central role of the auxin-signaling pathway, the auxin response factor (ARF) multigene family is present in all major divisions of land plants [4]. Considering the important role of ARF family members as regulators of plant growth and developmental processes, in the last few years there has been a considerable interest in studying the ARF family in both annual herbaceous plants [5, 6] and woody perennials [7, 8]. Characterization of ARF typically gives insights into the genomic structures [9], *loci* distribution across the genomes [10], sequence homology [11], phylogenetic history [12], and gene expression patterns [13] during development and/or biotic/abiotic stress. Efficient characterization of ARF family members could vastly improve both the number of useful genomic targets that could be identified to improve agronomic performance, and the speed with which they are characterized. Doing so within the legume family could have a dramatic impact on the availability of nutritious food and on ecosystem resilience [14].

Currently, the identification and characterization of plant gene families is laborious and time-consuming. It requires an iterative process of computational analysis to identify the gene family members of a given family. This process is based mainly on Basic Local Alignment Search Tool (BLAST) [15] searches interspersed with manual curation and pruning (Additional file 1: Figure S1). Due to the increasing number of sequences and the agronomical interest in plant gene families, this process could benefit from an automated tool. Although, numerous bioinformatics tools have been already developed to identify homologous proteins, these tools are largely restricted to static databases of fully sequenced animal genomes, and thus a tool that could work for plant genomes is needed.

To meet this need, we developed *geneHummus*, a novel R package that efficiently identifies members (accessions) of a plant gene family. By querying the SPARCLE and RefSeq databases, *geneHummus* can quickly isolate architecture identifiers from whole or draft genomes across taxonomic kingdoms, and update as new sequences are accessioned. In addition, *geneHummus* simplifies downstream analysis such as phylogenetic constructions and gene expression profiles. As a case study we focused on the auxin receptor factors gene (ARF) family in *Cicer arietinum* (chickpea) and other legumes, having previously manually annotated the ARF family in the chickpea genome [5], therefore having a gold standard dataset to which to compare our pipeline results. In our case study on the ARF gene family, the functionality of *geneHummus* allows an integrated workflow with phylogeny and expression profiles. We anticipate that our pipeline should be suitable for the study of any plant gene family, and likely other gene taxonomic families, vastly improving the speed and ease of genomic data processing.

### Implementation

**Fig. 2:**
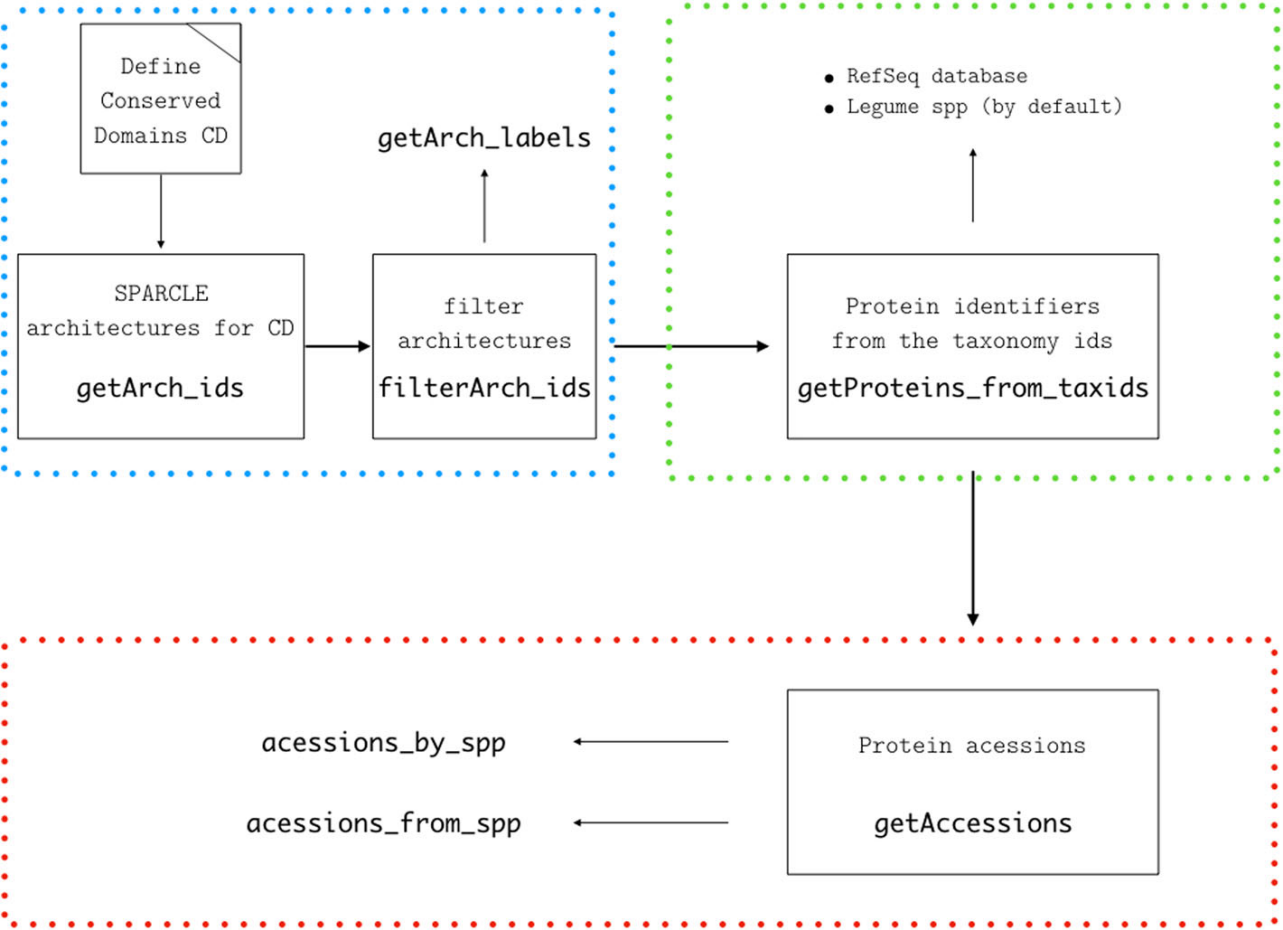
Workflow diagram for *geneHummus***.** The workflow shows the identification of protein families for legumes based on preparation of data (blue color), identification of family members by their electronic identifiers (green color), and retrieval of accessions and quantitative protein distribution *per* species (red color). R functions used in each step are highlighted in bold font

*geneHummus* is implemented as an R package and requires a minimal set of dependencies (*dplyr* [16], *stringr* [17], *rentrez* [18], httr, utils and curl packages) which are automatically downloaded from the CRAN repository [19] as needed. In this section, we describe the implementation of *geneHummus* in detail. There is also additional documentation describing the ARF case study available from our Github repository [20]. This pipeline was designed for a biologist end-user with a minimal amount of programming experience, using open source and free software (R, NCBI tools) to guide the user through the identification, characterization and expression analysis of gene families. Plant gene families are characterized by common protein structure. The structure that defines a given family is known from literature. For example, the hidden Markov model (HMM) profiles of the ARF gene family are the B3 DNA binding domain (B3), AUX_RESP, and AUX/IAA, which correspond with the conserved domains Pfam 02362, Pfam 06507, and Pfam 02309. The pipeline begins by defining the conserved domains accession numbers as a query against the SPARCLE database at the NCBI (Fig. 2). Then, you can get the SPARCLE architecture identifiers (ids) for each conserved domain and extract, or filter, only those architectures that characterize the ARF gene family. Next, the protein electronic ids for each candidate ARF architecture are retrieved. Depending on your dataset this step may take from seconds to 3–5 min. Note that if you have a very long list of protein ids, you may receive a 414 error when you try to interact with the NCBI E-utilities. *geneHummus* subsets the elements (up to 300 ids per list), so the functions can work properly. The retrieved protein ids are filtered by the taxonomy ids of interest [21], which in this case study were the legumes ids. *genehummus* returns only the protein ids hosted by the RefSeq database. Finally, the electronic ids are converted to protein accessions. At this point we have likely identified the whole set of ARF protein accessions from the legume family. Downloading the amino acid sequences from the accessions is straightforward. Once these sequences are downloaded, the relevant information may be used for phylogenetic and expression studies.

### Downstream analyses outside of the *geneHummus* R package

#### Phylogenetic analysis

Multiple protein sequence alignments (MSAs) were performed on the conserved Pfam domains [Pfam 02309: AUX/IAA family; Pfam 06507: ARF (AUX_RESP); Pfam 02362: B3 DNA binding domain (B3)] for the whole dataset. Multiple alignments were performed with MAFFT version 7.402 [22] using standard methods (FFT-NS-i) and the following parameters: mafft --thread 10 --threadtb 5 -- threadit 0 --reorder --leavegappyregion. A recent online version of the software is available as well [23]. A NJ tree was conducted using the JTT substitution model, 500 replicates of bootstrap, and pair-wise detection of gaps. Two representative of gymnosperms (*Picea sitchensis* and *Pinus pinaster*) were included as outgroup species.

#### Expression analysis

Using 1 single gene-model per locus, we created a BLAST database with the 24 ARF genes from the chickpea genome. Using Magic-BLAST [24], we studied the frequency of the ARF family in root tissues of two genotypes under drought stress and control conditions across 4 publicly available SRA libraries with the following parameters: alignment score = 125 bp, alignment identity ≥99% and read abundance in the four SRA libraries -- a list can be found at [25]. A normalization factor was estimated for each SRA library by dividing the average SRA size by the corresponding SRA size. The normalization factor was applied to each read to give normalized counts.

## Results and discussion

### External validation of data

**Fig. 3:**
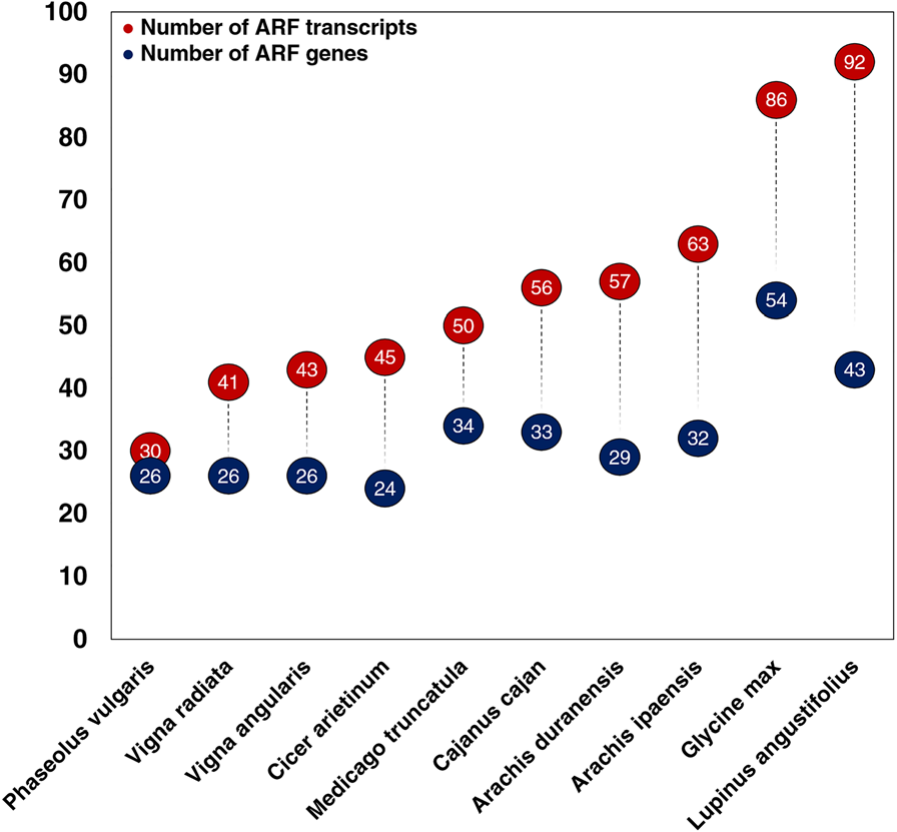
Total number of ARF sequences identified by CDART in legumes. Blue circles shows the number of ARF genes in each species of legumes, while red circles show the number of ARF transcripts

Numerous approaches have been developed to predict the function of different proteins. The Subfamily Protein Architecture Labeling Engine (SPARCLE; [26]), a recently developed resource by National Center for Biotechnology Information (NCBI), is one such approach. SPARCLE can help functional characterization of protein sequences by grouping them according to their characteristic domain architecture. We searched the SPARCLE database to obtain the whole set of molecular architectures based on the conserved domains that define the ARF gene family. Then, we filtered the data from SPARCLE to select the taxonomic group for the legume family (Fabaceae), and the source database (RefSeq; [27]). After these filters, we obtained over 560 different ARF legume proteins encoded by ~ 330 gene *loci* (Fig. 3). After separating these results by species, our pipeline identified 24 ARF proteins in the chickpea genome (*Cicer arietinum*), reproducing the results obtained previously with an iterative exhaustive BLAST search [5]. These results validate the *geneHummus* approach, which provides an automated way to produce these results in less than 6 min, as opposed to the exhaustive BLAST method which required significant manual curation and over 6 months of work. In addition, the *geneHummus* pipeline also returned the number of ARF proteins in 9 other legume species in this 6 min processing window (Fig. 3). Interestingly, the number of ARF proteins is similar in different species within the same genus (*Arachis duranesis* and *Arachis ipaensis*; *Vigna angularis* and *Vigna radiata*), as may be expected. In addition, species known to possess a high number of expanded paralogous genes due to whole-genome duplications events, such as *Glycine* and *Lupinus* lineages [28], showed the highest values both in the number of transcripts and number of ARF *loci*. We found that *geneHummus* is specific and sensitive by identifying the same sequences previously reported in other legume studies characterizing the ARF family through exhaustive searches, such as *M. truncatula* or soybean [6, 29]. But we also found more ARF accessions than had ever previously been reported, as *geneHummus* uses the latest updated RefSeq database version available at GenBank (Table 1). All of these results increase confidence in the validity of our approach. To find the ARFs in the different legume species using our pipeline, we developed an interactive shiny application to access this data [30]. After running the *geneHummus* pipeline, researchers interested in loading the relevant table for their gene families and taxa of interest can clone and modify [31] to easily share their results.

**Table 1:** Genome-wide identification of ARF gene family in plant species using exhaustive BLAST searches and *geneHummus* approaches

Species	Genome-wide analysis	*geneHummus*	
	Number of genes	Number of genes	Number of transcripts
*Arabidopsis thaliana*	22 [32]^a^	22	44
*Prunus persica*	18 [8]	17	27
*Medicago truncatula*	24 [6]	34	50
*Glycine max*	51 [29]	54	86
*Eucalyptus grandis*	17 [33]	17	27
*Brassica rapa*	31 [34]	34	48
*Solanum lycopersicum*	17 [35]	20	38
*Vitis vinifera*	19 [36]	19	31

^a^Okushima et al. reported 22 full-length ARF genes and one pseudo-gene

### Comparison with existing software

To date, a number of tools and pipelines are available to analyze sequences based on gene families. These tools are excellent for the identification of sequences in whole animal genomes; however, most of them cannot be applied to plant genomes or draft genomes, and have other limitations based on the databases they query and the software they use (see Table 2).

**Table 2:** Currently available ortholog identification tools

Tool	Purpose and Features	Platform
HomoloGene	● Constructs orthologous groups from the complete gene sets of 21eukaryotic species ● Includes only species with a complete genome or at least 10,000 UniGene entries	● Web interface
MultiMSOAR	● Identifies ortholog groups among multiple genomes ● Genome should be closely related	● Linux
OrthoMCL	● Groups proteins into ortholog groups based on their sequence similarity	● Galaxy server ● Linux
GeneSeqToFamily	● Finds orthologous genes and their corresponding gene families using the Ensembl Compara GeneTrees pipeline	● Galaxy server
OrthoFinder	● Identifies orthologous protein sequence families	● Linux
Ensembl Plants	● Utilizes reference genome sequences as a framework to integrate variant, functional, expression, marker, and comparative data for a number of plant species ● Ensembl plants does not include most legumes	● Web interface ● API
*geneHummus*	● Uses the Refseq Database, which is dynamically growing and manually curated ● Sequence data is streamed within cloud or local infrastructure so it doesn’t require downloading of genomic or protein sequences	● R ● Linux

*geneHummus* is a flexible tool that can work with almost any gene family and almost any plant species. HomoloGene [37] is an automated system for detecting homologs among 21 completely sequenced eukaryotic genomes; however, it is not flexible enough to be applied on draft genomes. MultiMSOAR [38, 39] and OrthoMCL [40, 41] are other tools that were developed to find ortholog groups among different genomes. These tools are limited to the software version and genomes included, which does not include the chickpea genome or other legume species. Ensembl plants [42] also provides programming tools to extract target genes families, but is similarly restricted to a limited number of genomes and the sequences that are hosted by the browser. While these tools are excellent for their intended purposes, they cannot meet the needs of plant geneticists interested in identifying conserved protein architectures.

*geneHummus* goes beyond existing tools in several ways. It identifies sequences for performing further analysis by searching for protein architectures from NCBI data, and then retrieving the gene family without requiring downloading of the gene or protein sequences. It uses the latest updated reference sequences, and is therefore more comprehensive in both the breadth of species and families covered for whole and partial genomes, as well as more accurate than static databases. Although *geneHummus* requires the end user to have a minimal familiarity with the R programming language, through the sequential call of four functions, the pipeline identifies the given gene family in a user-friendly and rapid environment. Provided that sequences are hosted by GenBank, the major advantage of *geneHummus* is that the user can apply it on the fly for any genome and it can be customized to be suitable for other agronomically important taxonomic families beyond legumes. For example, when installing the package, the user has access to several objects that contain the taxonomy ids for the families Brassicaceae, Cucurbitaceae, Rosaceae, and Solanaceae. This can be also customized for other families, which in practical terms makes *geneHummus* a useful tool for the plant research community. A summary of the above statements can be seen in Table 2.

### Phylogenetic analysis

**Fig. 4:**
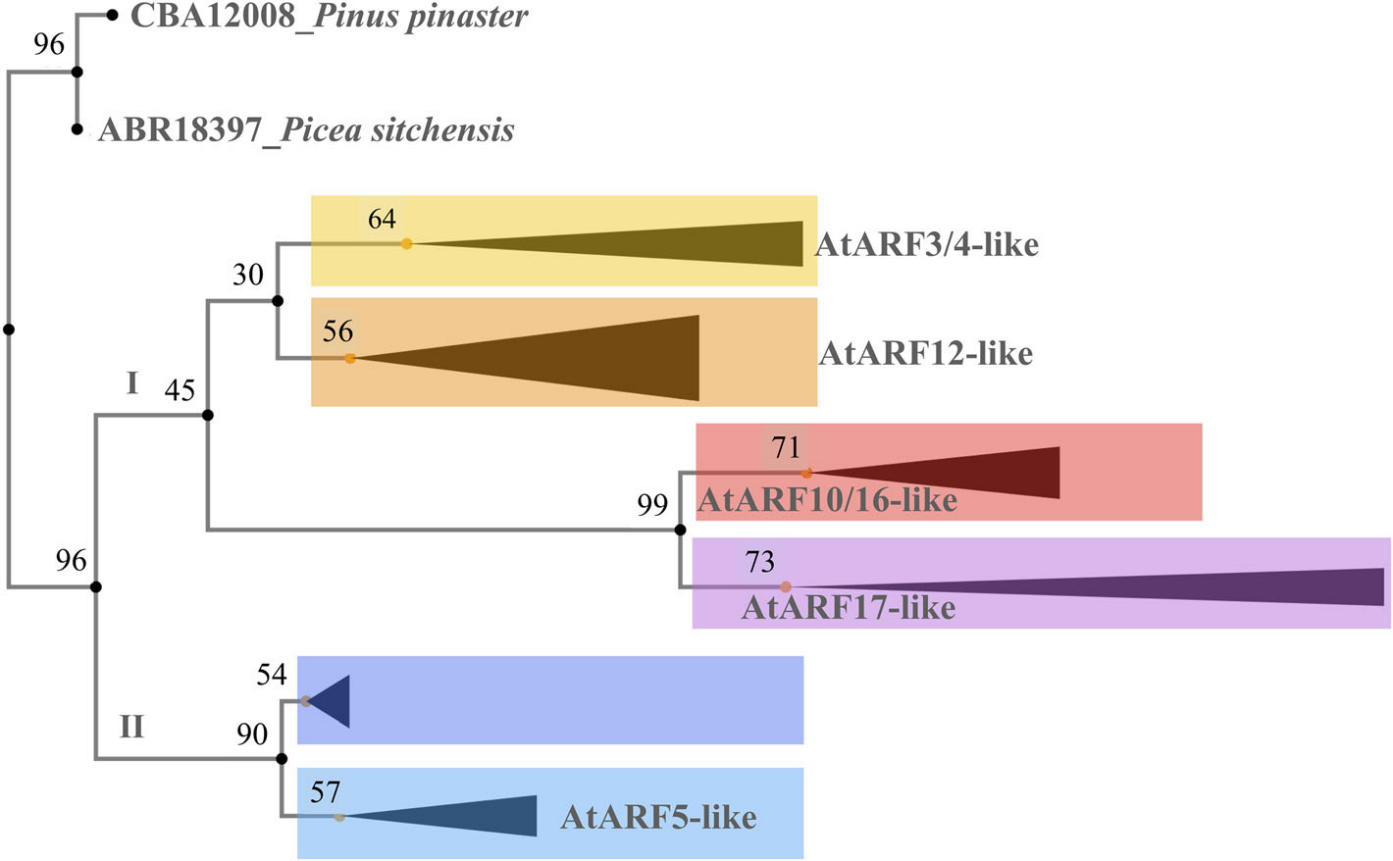
Analysis of phylogenetic relationships of ARF legume proteins. Phylogenetic analysis revealed two major clusters from the ARF lineage, one of which could be further subdivided into 6 clades The percentage of replicate trees in which the associated taxa clustered together in the bootstrap test (500 replicates) is shown next to the branches. Clades are named based on the phylogeny of the model plant *Arabidopsis thaliana*

Numerous downstream analyses and applications can be performed on *geneHummus* results. One example is phylogenetic analysis. Based on the conserved domains of ARF proteins, we explored and depicted the sequence relationships between the whole dataset. Two ARF proteins from the gymnosperm lineage were included as outgroup species. Gymnosperms have been resolved as the sister group of angiosperms. They diverged from their most recent common ancestor ~ 310 million years ago [43]. The phylogenetic distribution of the protein sequences revealed that all ARF sequences fall into two major groups (I and II) with well-supported bootstrap values (Fig. 4). The group I is the most numerous and may be further subdivided into clusters containing orthologs of the *Arabidopsis* sequences defining the well-known clades AtARF3/4-like, ARF12-like, ARF10/16-like, and ARF17-like [32]. Group II contain the cluster AtARF5-like. A second cluster in group II did not contain any *Arabidopsis* ortholog (data not shown) implying that this clade was derived through a long-term evolution for conserved functions across legume plant species. We labeled as sister pairs those proteins clustered together based on high bootstrap values (> 65%). Related to sister pairs involving chickpea, the phylogeny structures 8 sister pairs (seven pairs of *C. arietinum*-*M. truncatula* and one pair of *C. arietinum*-*L. angustifolius*). We did not observe any sister pair between two chickpea ARF proteins. This is an interesting evolutionary pattern. Chickpea diverged from *M. truncatula* ~ 10–20 million years ago [14]. Lack of chickpea sister pairs suggests that recent duplications (after chickpea and *Medicago* separated) have played a very limited role, if any, in the expansion of the ARF chickpea family, or that duplicated proteins did not change much since both species shared a common ancestor. Both hypothesis are plausible as well.

In addition, within the AtARF12-like group we observed a distinct clade made of 4 proteins based on bootstrap value, belonging to the ancestors of the cultivated peanut [44]. This suggests that these are orthologs of an ancestral gene emerged after the speciation of *Arachis* genus (Additional file 2: Figure S2). We are looking forward to the peanut genome becoming public so we can validate these results.

### Expression profiles for gene discovery

**Fig. 5:**
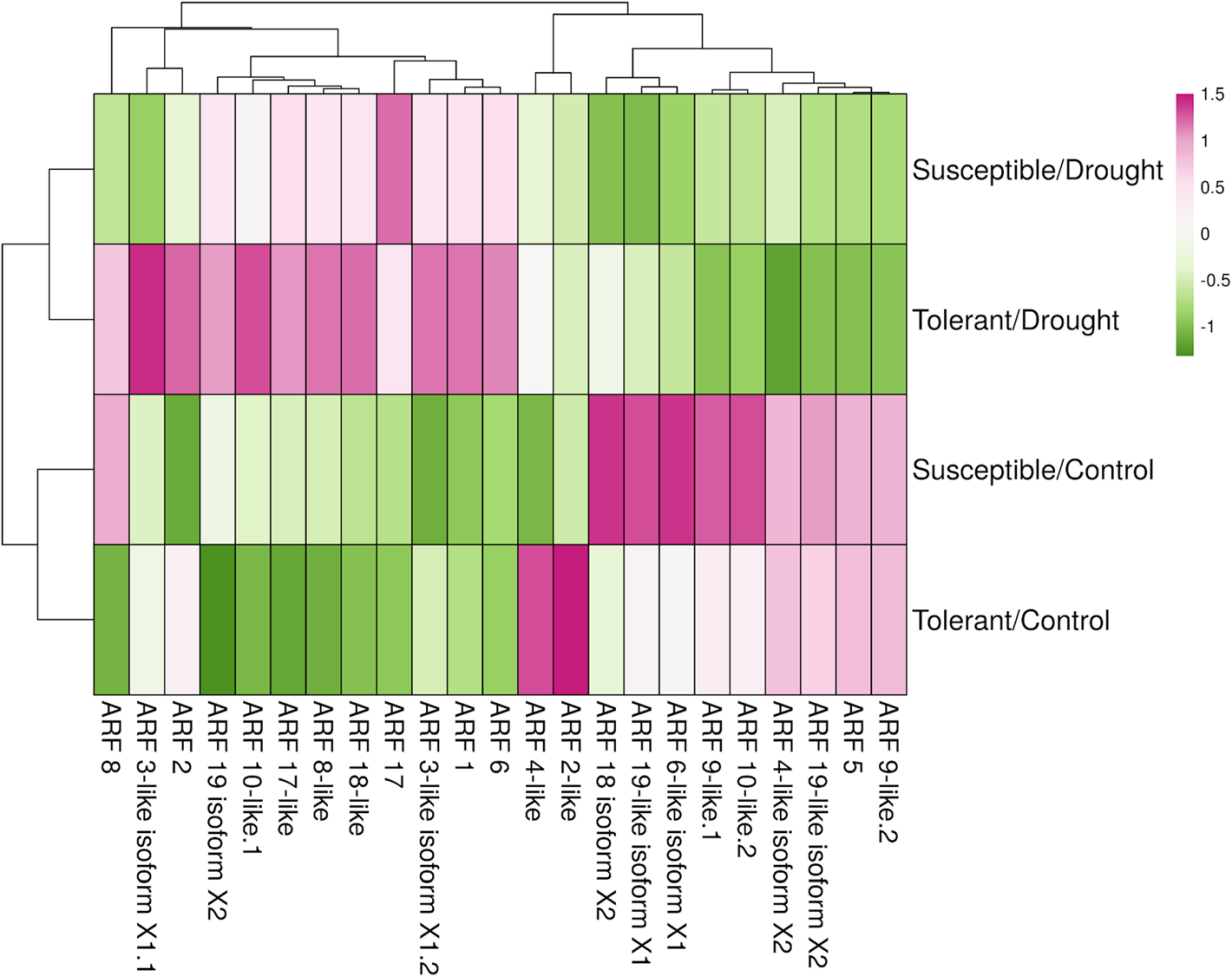
Differential abundance of ARF gene *loci* in *Cicer arietinum* under drought or normal conditions. Drought samples are clustered on the top, while control samples are clustered on the bottom

Another useful application that *geneHummus* results could be used for is identifying genes of interest in transcriptomic experiments and their role in responding to environmental perturbations. We used the genes identified by *geneHummus* as a reference database to study their expression in drought conditions using freely available SRA data and the Magic-BLAST tool [24]. We identified datasets in the SRA that isolated sequences from the root of the chickpea plant grown in either drought or control conditions, and was identified as belonging to either a tolerant/drought or susceptible/drought strain. Upon analysis, we identified 3 transcripts that were differentially expressed in the drought-tolerant strain in drought conditions as opposed to the drought-susceptible strain (Fig. 5). This ARFs could be important targets for the genetic improvement of chickpea via conventional breeding or biotechnological approaches.

## Conclusions

Applying the *geneHummus* pipeline, we characterized the structure and phylogeny of the whole ARF proteins dataset in the legume family. As a case study, we also characterized the expression profile of the gene family in *Cicer arietinum.* The utility of this pipeline stems from a reduction in hands-on annotation time combined with high specificity and sensitivity in extracting proteins from the RefSeq database and providing interaction with the suite of other NCBI tools such as the conserved domain architectures based on the recently developed NCBI resources SPARCLE and the aligner Magic-BLAST. *geneHummus* is a powerful tool for the identification of gene family sequences that could be used in phylogenetic analysis. Our results indicates that most proteins are very well conserved across genera, with abundant multi-species clades. This suggests that these proteins are involved in common basic cellular actions. This orthology information could be used to infer the function of a previously uncharacterized protein in a given species based on the known function of the protein in another genera. This is a particularly strong approach for comparative genomics. Once the sequences have been identified, given the ability of available SRA libraries for a number of tissues and conditions, the user can get the most out of the pipeline by using Magic-BLAST-based differential expression analysis to identify genes of interest for certain conditions. This tool will help investigators discover genes, and has particular applicability to plant breeding programs, among other applications. Our pipeline has been suitable for ARF family detection on all plant Genomes tested, and should be suitable for other gene families, vastly improving the speed and ease of genomic data processing.

## Availability and requirements

Project name: *geneHummus.*

Project home page: https://github.com/NCBI-Hackathons/GeneHummus

Operating system(s): Platform independent.

Programming language: R.

Other requirements: R 3.5.0 or higher.

License: MIT license.

Any restrictions to use by non-academics: None.

## Additional files

Additional file 1: **Figure S1.** Approaches for identification of gene families. **a**. Exhaustive identification based on BLAST searches. Sequences from a close-related species are used as queries using BLAST to identify the corresponding gene members in a target genome. For validation, sequences from a second model organism are also commonly used as queries following the same procedure. The hidden Markov model profiles of the gene family is used to confirm the identity of the candidate genes. **b.** Automatic pipeline implemented in *geneHummus*. The defining conserved domains of the gene family are used to parse the SPARCLE database and retrieve sequences grouped by a given protein architecture. (JPEG 199 kb)

Additional file 2: **Figure S2**. Cluster from clade AtARF12-like containing *Arachis* specific ARFs. (TIFF 1501 kb)

## Data Availability

The datasets generated and/or analysed during the current study are available in the *geneHummus* repository, https://github.com/NCBI-Hackathons/GeneHummus

## Abbreviations

ARF: Auxin receptor factor
BLAST: Basic Local Alignment Search Tool
HMM: Hidden Markov model
MSA: Multiple sequence alignment
NCBI: National Center for Biotechnology Information
NGS: Next-generation sequencing
SPARCLE: Subfamily Protein Architecture Labeling Engine
SRA: Sequence Read Archive
