# Supplementary figures and images for: geneHummus: an R package to define gene families and their expression in legumes and beyond
Source: BMC Genomics. 2019 Jul 18;20:591. doi: 10.1186/s12864-019-5952-2 (PMC6639926; doi:10.1186/s12864-019-5952-2)

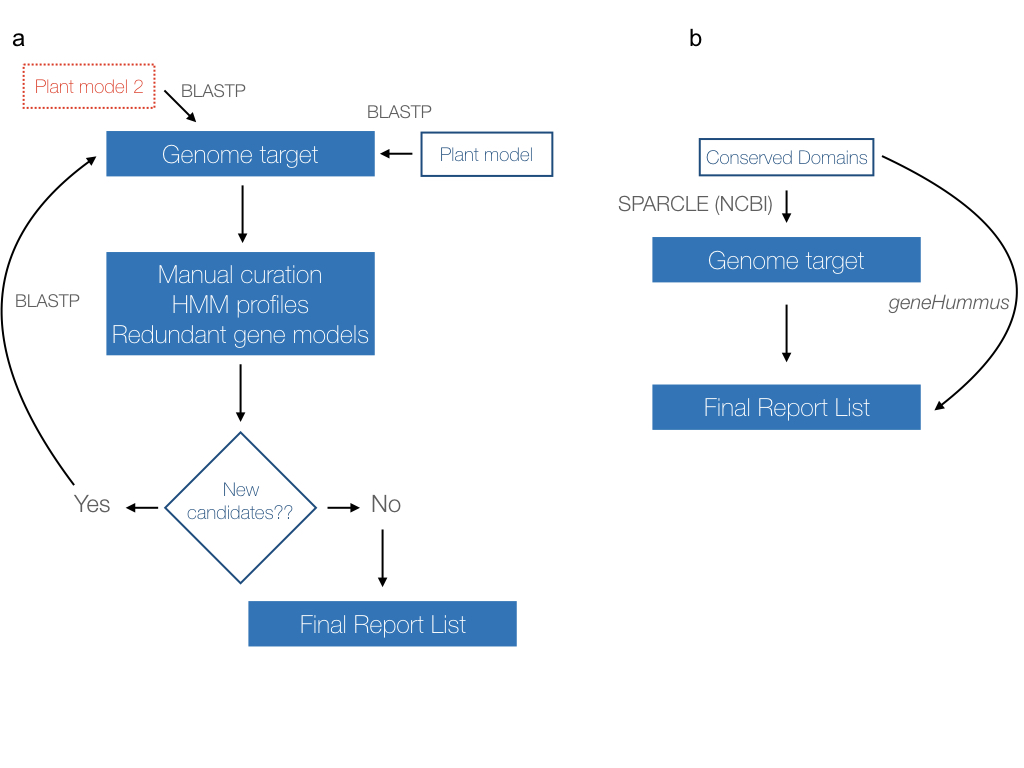

Supplement: Supplementary file 1 — Figure S1. Approaches for identification of gene families. a. Exhaustive identification based on BLAST searches. Sequences from a close-related species are used as queries using BLAST to identify the corresponding gene members in a target genome. For validation, sequences from a second model organism are also commonly used as queries following the same procedure. The hidden Markov model profiles of the gene family is used to confirm the identity of the candidate genes. b. Automatic pipeline implemented in geneHummus. The defining conserved domains of the gene family are used to parse the SPARCLE database and retrieve sequences grouped by a given protein architecture. (JPEG 199 kb) [file 12864_2019_5952_MOESM1_ESM.jpeg]

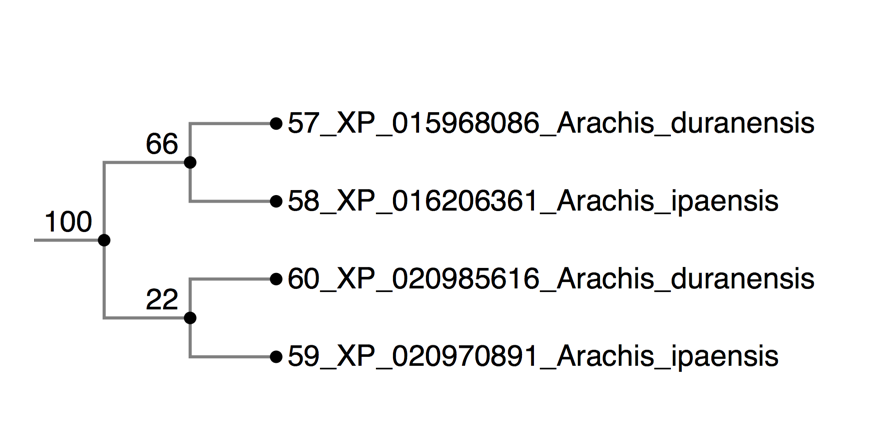

Supplement: Supplementary file 2 — Figure S2. Cluster from clade AtARF12-like containing Arachis specific ARFs. (TIFF 1501 kb) [file 12864_2019_5952_MOESM2_ESM.tiff]
